# Blood pressure tables for Chinese adolescents: justification for incorporation of important influencing factors of height, age and sex in the tables

**DOI:** 10.1186/1471-2431-14-10

**Published:** 2014-01-16

**Authors:** Xuejin Jiang, Zhongqiang Cao, Lijun Shen, Jing Wu, Zhongliang Li, Jing Gao, Youjie Wang

**Affiliations:** 1 Department of Maternal and Child Health, School of Public Health, Tongji Medical College, Huazhong University of Science & Technology, HangKong Road 13, Wuhan, China

**Keywords:** Adolescent, Hypertension, Blood pressure table

## Abstract

**Background:**

Elevated blood pressure (BP) in childhood was a predictor of hypertension in adulthood and contributes to the current epidemic of cardiovascular disease. It is necessary to identify abnormal BP in children and adolescents with accurate BP tables based on several crucial factors. The purpose of this study was to identify the important influencing factors of BP of Chinese adolescents.

**Methods:**

BP, height, and body weight were assessed in 32221 normal-weight Chinese adolescents aged 12–17 years. An equal number of 6815 subjects from boys and girls were individually matched by height and age to assess the independent effect of sex on BP; and an equal number of 1422 subjects from each of the age groups (12, 13, 14, 15, 16 and 17 years) were individually matched by sex and height to estimate the independent effect of age on BP. Height of each sex and age was divided into eight height groups - ~5^th^, ~10^th^, ~25^th^, ~50^th^, ~75^th^, ~90^th^, ~95^th^, and 95^th^ ~ percentiles- and the Spearman’s correlation between height percentiles and BP was used to examine the independent effect of height on BP.

**Results:**

Boys had higher systolic BP (SBP) and diastolic BP (DBP) than girls after controlling for age and height. BP increased with age after controlling for sex and height. In each age group, both SBP and DBP increased alongside increasing height in boys and girls.

**Conclusions:**

Sex, age and height are all independent determinants for BP levels in Chinese adolescents. It is essential to incorporate these three factors for the establishment of the BP reference tables.

## Background

Hypertension in children and adolescents has become crucial health issues since its increasing prevalence [1,2]. It has been previously reported that the incidence rate of hypertension and prehypertension in Chinese adolescents aged 12–17 years is 3.1 and 7.2, respectively [3]. A number of studies have shown that blood pressure (BP) in adolescents tends to track from childhood into adulthood [4-7]. Thus, the development of a BP table to identify hypertension or prehypertension in children and adolescents is necessary for the screening, detection, and diagnosis of these conditions in the pediatric population. Hansen et al. revealed that hypertension and prehypertension in children and adolescents are frequently underdiagnosed, and suggested that this low rate of diagnosis was caused by clinicians’ lack of knowledge of normal BP ranges in the pediatric population [8]. Specifically, sex and the ever-changing biometric factors intrinsic to the growing children (e.g., age, height) cause the BP cutoff for hypertension in children and adolescents to be more difficult to determine than that for adults, as laid out in the standard-setting *Fourth Report on the Evaluation of the Diagnosis*, *Evaluation*, *and Treatment of High Blood Pressure in Children and Adolescents* published by the U.S. National High Blood Pressure Education Program (NHBPEP) [9]. In this publication, hundreds of normal and abnormal BP values based on sex, age and height percentiles are listed in the BP table for the evaluation of BP in children and adolescents, providing a precise classification of BP. However, so many normal and abnormal BP cutoffs exist, which are difficult for physicians or pediatricians to remember; likewise, it is time-consuming for clinicians to look up all of the appropriate tables, and these tables may not even be available in certain clinic settings. Furthermore, these U.S.-based data may not be accurately applied to children and adolescents in other parts of the world because BP level and prevalence of hypertension vary in different racial and ethnic groups [10,11].

In recent years, several countries have attempted to develop their own BP tables for diagnosing hypertension in children and adolescents. In 2010, the BP table for evaluating and diagnosing childhood hypertension was included in the Chinese guidelines for the management of hypertension; these guidelines were based on the composite data of nine large Chinese epidemiological studies involving 112227 children and adolescents hypertension ages 3–18 years old [12]. Only sex and age were considered in this Chinese BP reference making this table simpler to use than those published in the NHBPEP report [9]. It has been previously reported that body size, physiological maturation, and age are the major determinants of children and adolescents BP [13-15], but this Chinese report did not explain the reasons for the incorporation of sex and age only rather than sex, age and height simultaneously, suggesting that it is debatable whether or not this Chinese BP reference can be accurately applied to the BP evaluation of Chinese children and adolescents. In present research, we used a large sample to investigate whether a BP table that only considered sex and age (i.e., the Chinese model) could accurately predict hypertension and prehypertension among Chinese children and adolescents as well as or better than a BP table that simultaneously considered sex, age, and height (i.e., the NHBPEP model).

## Methods

### Study subjects

The subjects of this study were recruited from Changsha city, the capital city of Hunan province of China. All junior and senior high school students in Changsha underwent a medical examination by a trained pediatrician or nurse in 2008, and all the students and their parent or legal guardian signed the informed consent. Among them, 38317 adolescents aged 12–17 years old were initially included in the study. As body weight is an important determinant of BP for children and adolescents, and if overweight and obese children and adolescents are included in the normative database the norms for BP will increase as the increasing prevalence of overweight and obesity among children and adolescents. We excluded from our analysis 6096 adolescents who were overweight or obese based on the Body Mass Index Reference Norm for Screening Overweight and Obesity for Chinese Adolescents [16]. In total, 32221 adolescents, of whom 14999 were boys and 17222 were girls, were included in our study analysis.

This study ethics was approved by the research ethics committee of Tongji Medical College, Huazhong University of Science and Technology.

### Body weight, height, and BP measurements

All measurements were conducted in a quiet clinical setting by trained professionals. Height (cm) and body weight (kg) were measured to the nearest 0.1 cm and 0.1 kg respectively using an electronic height-weight measurement instrument (Shuangjia, Shenzhen, China) that had been adjusted before use. All subjects were required to stand straight without shoes and with their arms hanging relaxed, and to wear thin clothes [17].

BP was measured to the nearest 2 mmHg using a mercury sphygmomanometer (Yuyue, Jiangsu, China) with a cuff appropriate to the size of the child’s upper right arm. Students were asked to relax and rest for 5 minutes before BP measurement and to keep sitting with the arm at the level of the heart during the measurement process [17]. Systolic BP (SBP) was defined as the onset of the first Korotkoff phase, and diastolic BP (DBP) was defined by the fourth Korotkoff phase. We adopted the fourth Korotkoff sound for DBP in this study because it is more reliable and reproducible than five Korotkoff, and it was easy for us to control the measurement error in our large sample size [18,19]. BP was measured 3 times and the mean value was used for our assessment.

### Matching and grouping

Because the relationships of BP with both age and height are nonlinear, the conventional regression model is not suitable to investigate effect of each variable (sex, age and height) on BP [20]. In the present study, we used a matching and a grouping technique to examine the role of sex, age, and height in BP levels, respectively. To assess the effect of sex on BP, each boy was regarded as a potential subject and was individually and randomly matched to a girl of the same age and same height (within ± 1.0 cm). To evaluate the role of age on BP, each student in the 12-year-old group was regarded as a potential subject and was individually and randomly matched to 5 subjects from the 13- to 17-year-old groups with the same sex and same height (within ± 1.0 cm). Height of each sex and each age group was divided into eight height groups - ~5^th^, ~10^th^, ~25^th^, ~50^th^, ~75^th^, ~90^th^, ~95^th^, and 95^th^ ~ percentiles- to estimate its influence on BP.

### Statistical analysis

SPSS Statistics 11.0 (SPSS, Chicago, IL) and Microsoft Excel 2007 software were used for statistical analysis. Descriptive statistics for height, SBP and DBP were calculated for all age groups in each sex and expressed as mean ± s.d. Before matching, the differences in BP mean values between boys and girls were determined using Student’s *t*-test; and the differences in BP among six age groups were tested using one-way analysis of variance, and between-group differences were analyzed post-hoc using the SNK-q method. After matching, Paired *t* test was used to compare the differences in BP between boys and girls; and two-way analysis of variance was used to test the differences in BP among six age groups, and SNK-q method was used to analyze between-group differences. In order to investigate the effect of height on BP, Spearman’s correlation analysis was used to assess the relationship of height percentile with BP.

## Results

A total of 32221 normal-weight students aged 12–17 years old who underwent a medical examination in 2008 were included in our analysis. Table 1 summarizes the mean height, SBP, and DBP by sex and age, this table shows that both SBP and DBP increased significantly with age throughout all age groups except the differences of BP between 15 and 16 years age groups of girls was not statistical significant for SBP (p = 0.118) and DBP (p = 0.258); boys had a significantly higher SBP than girls in all age groups (p < 0.001), and had a higher DBP than girls in all age groups, but this was not statistical significant for 12-year-olds (p = 0.233).

**Table 1:** **Height**, **systolic and diastolic blood pressure according to sex and age** (**mean** ± **s.d**.)^**1**^

**Age**	**N**		**Height**		**p value**^**$**^	**SBP**		**p value**^**$**^	**DBP**		**p value**^**$**^
	**Boys**	**Girls**	**Boys**	**Girls**		**Boys**	**Girls**		**Boys**	**Girls**	
12	1022	1304	154.4 ± 8.2	153.8 ± 6.1	0.052	99.7 ± 8.3	98.2 ± 8.0	<0.001	64.2 ± 5.3	63.9 ± 5.5	0.233
13	1690	1965	161.0 ± 8.1	156.5 ± 5.6	<0.001	102.1 ± 9.2	99.0 ± 8.1	<0.001	65.4 ± 6.1	64.4 ± 5.6	<0.001
14	2717	3039	165.6 ± 7.0	157.8 ± 5.3	<0.001	104.6 ± 9.5	99.7 ± 8.3	<0.001	66.7 ± 6.4	65.1 ± 6.0	<0.001
15	3233	3823	168.8 ± 6.3	158.6 ± 5.4	<0.001	107.4 ± 9.4	100.7 ± 8.4	<0.001	68.2 ± 6.6	65.7 ± 6.1	<0.001
16	3491	4274	170.2 ± 6.0	158.8 ± 5.4	<0.001	109.6 ± 9.8	101.1 ± 8.4^▼^	<0.001	69.4 ± 6.8	65.9 ± 6.1^▼^	<0.001
17	2846	2817	170.7 ± 6.1	159.0 ± 5.4	<0.001	110.5 ± 9.7	102.2 ± 8.8	<0.001	70.0 ± 6.9	66.3 ± 6.1	<0.001

^1^Abbreviations are as follows: *SBP* systolic blood pressure; *DBP* diastolic blood pressure.

^▼^P > 0.05, vs. 15 years group.

^$^*T*-test.

We matched the age and height to examine the relationship between sex and BP level. Among the 32221 subjects, 6815 boys and 6815 girls were successfully matched with subjects of the same age and same height. As shown in Table 2, after controlling for age and height, we found that boys still had a significantly higher SBP than girls in all age groups (p < 0.05). Boys also had a higher DBP than girls in all age groups, but this was not statistical significant for 12-year-olds (p = 0.298) and 13-year-olds (p = 0.532).

**Table 2:** **Systolic and diastolic blood pressure of students in each age group after matched by height** (**mean** ± **s.d**.)^**1**^

**Age**	**N**		**SBP**		**p value&**	**DBP**		**p value&**
	**Boys**	**Girls**	**Boys**	**Girls**		**Boys**	**Girls**	
12	887	887	99.4 ± 8.1	98.1 ± 8.0	<0.001	64.1 ± 5.2	63.8 ± 5.7	0.298
13	1181	1181	101.0 ± 8.8	99.2 ± 8.2	<0.001	64.7 ± 5.8	64.6 ± 5.6	0.532
14	1402	1402	103.3 ± 9.3	100.2 ± 8.4	<0.001	66.0 ± 6.4	65.5 ± 6.1	0.041
15	1316	1316	106.0 ± 9.3	101.1 ± 8.3	<0.001	67.5 ± 6.5	65.8 ± 6.0	<0.001
16	1193	1193	108.7 ± 9.8	101.4 ± 8.8	<0.001	68.7 ± 6.7	65.9 ± 6.0	<0.001
17	836	836	110.3 ± 9.8	103.0 ± 9.3	<0.001	69.8 ± 7.0	66.7 ± 6.1	<0.001

^1^Abbreviations are as follows: *SBP* systolic blood pressure; *DBP* diastolic blood pressure.

^&^Paired *T*-test.

We found height to be closely related with age during puberty. To assess the independent effect of age on BP, we matched sex and height among subjects in the 12- to 17-year-old age groups. Of the 32,221 subjects, 361 boys and 1,061 girls in each age group were successfully matched by height. Table 3 shows that after height-matching, although the effect of age on BP was attenuated, age was still significantly associated with both SBP (p < 0.001) and DBP (p < 0.001).

**Table 3:** **Systolic and diastolic blood pressure of students in each sex after matched by height** (**mean** ± **s.d**.)^**1**^

**Sex**		**Age**						p value**
		**12**	**13**	**14**	**15**	**16**	**17**	
Boys	SBP	102.5 + 8.5	103.8 + 8.8^▲^	104.2 + 9.3^★^	106.1 + 9.3	108.7 + 9.8	110.4 + 9.7	<0.001
	DBP	65.3 + 5.7	66.0 + 6.3^▲^	66.5 + 6.6^★^	67.3 + 6.7^◆^	68.9 + 6.3	69.4 + 6.7^■^	<0.001
	N	361	361	361	361	361	361	
Girls	SBP	98.6 + 8.1	98.9 + 8.1^▲^	99.3 + 8.1^▲★^	100.5 + 8.2	100.9 + 8.4^▼^	101.4 + 8.8^■^	<0.001
	DBP	64.1 + 5.6	64.3 + 5.6^▲^	64.8 + 5.8^★^	65.3 + 5.7	65.9 + 6.3	66.0 + 6.0^■^	<0.001
	N	1061	1061	1061	1061	1061	1061	

^1^Abbreviations are as follows: *SBP* systolic blood pressure; *DBP* diastolic blood pressure.

^▲^P > 0.05, vs. 12 years group; ^★^P > 0.05, vs. 13 years group; ^◆^P > 0.05, vs. 14 years group; ^▼^P > 0.05, vs. 15 years group; ^■^P > 0.05, vs. 16 years group.

**Two-way analysis of variance.

**Figure 1:**
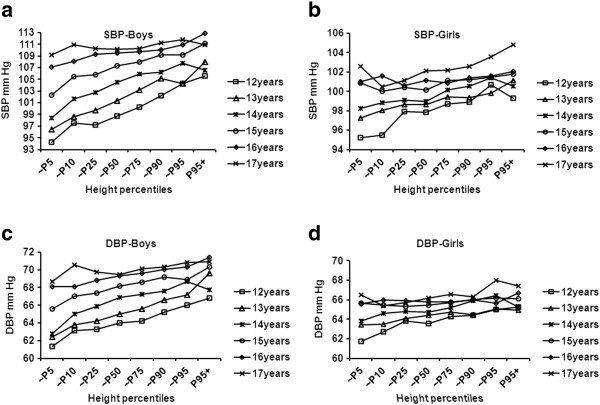
**Height percentiles and mean systolic and diastolic blood pressure by sex for six age groups.** Graph of Systolic blood pressure (SBP) in boys **a)** and in girls **b)**, diastolic blood pressure (DBP) in boys **c)** and in girls **d)**. SBP and DBP increased with height in each age group among boys and girls. Height percentiles in each age group were closely associated with SBP and DBP (p < 0.01 or p < 0.05) in both boys and girls.

We calculated the age- and sex-specific BP level in different height percentile groups. Figure 1 shows that SBP and DBP increased with height in each age group among boys and girls. Spearman’s correlation analysis showed that the height percentiles in each age group were closely associated with SBP and DBP (p < 0.01 or p < 0.05) in both boys and girls, respectively. This suggests that height is an independent predictor of BP after controlling for sex and age.

## Discussion

It is well-established that adult hypertension is the result of a process that starts early in life [4-7]. Measuring BP in children and adolescents at every medical examination is critical to the early detection and prevention of adult cardiovascular diseases. We argue that BP measurement should be interpreted based on childhood normative data of BP. The BP of children and adolescents increases with age and body size, making it impossible to use a single BP level to define hypertension, as is done in adults.

The most widely used diagnostic criterion for elevated BP in children and adolescents, as published by the NHBPEP, was based on 10 studies involving more than 70000 adolescents aged 1 to 17 years old. The BP criteria based on sex, age and height provide accurate cutoff values of hypertension and prehypertension in children and adolescents. However, on account of difference of BP level across the world, the BP reference norms established for one particular population may not be applicable to other. Then, some local BP standards based on sex, age and height were developed in several countries and regions (e.g., Germany, Korea, and European) [21-23]. BP normograms only based on sex and age were also established in some countries (e.g., China, Britain); however, those reports did not provide the reasons for not considering height.

Using the data analyzed in our study, we found that boys had a higher SBP and DPB than girls in each age group regardless of height-matching. We also found that SBP and DBP increased with age before and after controlling for subjects’ sex and height. After controlling for sex and age, both SBP and DBP increased with height. Our results strongly showed that sex, age and height are independent influence factors of BP and indicated that sex, age and height play important roles in determining BP of Chinese adolescents.

Although the BP tables for children and adolescent published in the NHBPEP report provide a reasonable basis for diagnosing hypertension in children and adolescents, accurate diagnosis is complicated. There exist hundreds of hypertension cut-offs in children based on the BP percentile for sex, age, and height, and these cutoffs are difficult for pediatric clinicians to remember and, at times, access. As a result, hypertension is frequently underdiagnosed in children and adolescents [8].

Many investigators have tried to simplify the adolescents BP cutoff tables to make them easy and memorable. Wang et al. suggested that BP percentile charts can be simplified by establishing a normal percentile based solely on height for each sex group in Chinese children aged 7–10 years old as their studies demonstrated that age has little impact on BP levels once height is taken into consideration [24]. However, this phenomenon was not found in Chinese adolescents aged 12–17 years old in the present study. We found that age was still significantly associated with both SBP and DBP although the effect of age on BP was attenuated after taken height into account. The differences may be possibly owing to physiological maturation and hormonal changes occurring in the body during puberty. Based on the existing BP tables published by NHBPEP according to sex, age and height, some simple BP tables were established using different methods. Kaelber et al. developed a simplified BP screening table from using systolic and diastolic thresholds as the lowest abnormal BP values in the prehypertensive range, regardless of height percentile [25]. Badeli et al. recommended a simpler table of formulas consisting of age and the 90th percentiles of blood pressure for the 5th percentiles of height [26]. These simplified tables have a very high sensitivity for identifying all abnormal pediatric BP values. However, they are likely to overdiagnose some high-statured children and adolescents as having hypertension or prehypertension. Then, more accurate and simpler BP tables for children and adolescents are needed to be developed in the future in order to precisely and easily identify children and adolescents who have abnormal BP. Of course, the simplified BP tables should be based on the accurate and precise tables established according to the crucial influencing factors.

Among other concerns, hypertension is associated with significant organ damage and morbidity. Accurate BP screening and appropriate diagnostic evaluation is critical throughout the lifespan. In this study, we found that sex, age, and height were independently associated with BP in Chinese adolescents. The present study suggests that it is essential to consider these three factors simultaneously for establishing accurate BP tables for Chinese adolescents.

## Conclusions

Our study found that sex, age and height were independent influencing factors of BP level in Chinese adolescents. The findings of this study strongly suggest that it is necessary to take these three factors into consideration simultaneously for establishing accurate BP reference tables for Chinese adolescents.

## Abbreviations

BP: Blood pressure; SBP: Systolic blood pressure; DBP: Diastolic blood pressure.

## Competing interests

The authors declare that they have no competing interests.

## Authors’ contributions

Conception and design: XJJ and YJW. Analysis and interpretation of data, manuscript: XJJ. Data extraction: XJJ, ZQC, LJS, JW, ZLL, JG. Revising article critically for important intellectual content: YJW. All authors read and approved the final manuscript.

## Pre-publication history

The pre-publication history for this paper can be accessed here:

http://www.biomedcentral.com/1471-2431/14/10/prepub
